# Polystyrenesulfonate-catalyzed synthesis of novel pyrroles through Paal-Knorr reaction

**DOI:** 10.1186/2191-2858-2-11

**Published:** 2012-03-27

**Authors:** Mandira Banik, Bianca Ramirez, Ashwini Reddy, Debasish Bandyopadhyay, Bimal K Banik

**Affiliations:** 1 Department of Chemistry, The University of Texas-Pan American, 1201 West University Drive, Edinburg, TX 78539, USA

**Keywords:** pyrrole, polystyrene sulfonate, Paal-Knorr reaction, catalysis.

## Abstract

**Background:**

The classical Paal-Knorr reaction is one of the simplest and most economical methods for the synthesis of biologically important and pharmacologically useful pyrrole derivatives.

**Results:**

Polystyrenesulfonate-catalyzed simple synthesis of substituted pyrroles following Paal-Knorr reaction has been accomplished with an excellent yield in aqueous solution. This method also produces pyrroles with multicyclic polyaromatic amines.

**Conclusions:**

The present procedure for the synthesis of *N*-polyaromatic substituted pyrroles will find application in the synthesis of potent biologically active molecules.

## Background

**Figure 1:**
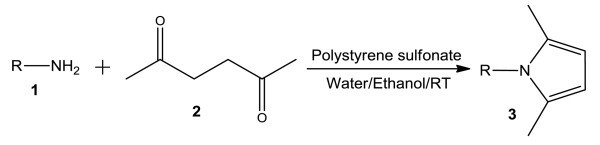
**Polystyrene sulfonate-catalyzed simple synthesis of *N*-substituted pyrroles**.

Pyrroles have demonstrated important different biological activities in several areas [1]. On this basis, diverse methods for the synthesis of substituted pyrroles are known [2]. For example, Conjugate addition reaction has been developed for the preparation of pyrroles [3]. Pyrroles can also be prepared from transition metals [4], reductive coupling reaction [5], aza-Wittig reaction [6], and other multi-step reactions [7]. However, Paal-Knorr reaction is the most reliable methods for the synthesis of pyrroles [8]. Clay-induced [9] reaction and microwave irradiation method [10] have been used for the synthesis of pyrroles. Several synthetic procedures from our laboratory have also been reported [11-16]. In this article, we report simple synthesis of substituted pyrroles using an aqueous solution of polystyrenesulfonate in ethanol. Unlike many methods, synthesis of pyrroles in aqueous solution is new and challenging (Figure 1).

## Results and discussion

Synthesis of pyrroles with polyaromatic amines has not been reported. High-power microwave irradiation or considerable amounts of acids under anhydrous conditions are always necessary in the Paal-Knorr reaction. Therefore, mild reaction conditions that can overcome some of the shortcomings of previous methods are necessary. In continuation of our research on environmentally benign reaction and biological evaluation of various polyaromatic compounds as novel anticancer agents [17-22], we have investigated Paal-Knorr reaction using aqueous polystyrenesulfonate. After various experimentations, we have identified polystyrenesulfonate as a good catalyst for the preparation of pyrroles starting from amines and 1,4-diketo compound. Several amines including monocyclic, bicyclic, tricyclic, and tetracyclic aromatic amines were used. The other starting material was commercially available 2,5-hexanone (acetonylacetone) (Figure 1). At the beginning of the procedure, the diketo compound (**2**), the amine (**1**) and polystyrenesulfonate were added in ethanol. The mixture was then stirred at room temperature for 2 h-overnight depending upon the nature of the aromatic amines. The reaction mixture was basified with aqueous sodium bicarbonate solution and extracted with dichloromethane. The organic layer was then washed with brine, dried with sodium sulphate and evaporated. The yields of the products are shown in the Table 1. The less basic aromatic amines needed longer reaction time although the yields are comparable to the more basic amino compounds.

**Table 1:** Polystyrene sulfonate-catalyzed simple synthesis of *N*-substituted pyrroles following Figure 1

Entry	Amine	Product	Time (h)	Yield (%)^a^
1			10	96
2			15	81
3			11	83
4			19	98
5			18	94
6			20	88
7			22	85

^a^isolated yield

## Conclusions

In conclusion, a new procedure for the synthesis of *N*-substituted pyrroles has been developed. Because of the simplicity of the procedure, products can be isolated very easily. The compounds reported herein will be tested against a number of cancer cells *in vitro*. This reaction will be applicable to the synthesis of various organic compounds of medicinal interests.

## Methods

### General

FT-IR spectra were registered on a Bruker IFS 55 Equinox FTIR spectrophotometer as KBr discs. ^1^H NMR (600 MHz) and ^13^C-NMR (150 MHz) spectra were obtained at room temperature with Bruker-600 equipment using TMS as internal standard and CDCl_3 _as solvent. Analytical grade chemicals (Sigma-Aldrich Corporation) were used throughout the project. Deionized water was used for the preparation of all aqueous solutions.

### General procedure for the synthesis of pyrroles (3)

Amine (1.0 mmol), 2,5-hexanedione (1.2 mmol) and polystyrene sulfonate (18 wt. % solution in water) in water/ethanol (1:1) mixture was stirred at room temperature as specified in Table 1 and the progress of the reaction was monitored by TLC every 30 min. After completion of the reaction (Table 1) the reaction mixture was basified with aqueous sodium bicarbonate solution and extracted with dichloromethane. The organic layer was then washed with brine, dried with sodium sulphate and evaporated to isolate the pure product.

## Competing interests

The authors declare that they have no competing interests.

## Authors' contributions

MB performed the reactions with the help of BR and AR. DB advised to use the catalyst with some initial help to run the project. All authors read and approved the final manuscript.

## Authors' information

MB is a high school research participant; BM is an undergraduate research participant and AR is a graduate student.
